# Lycopene enhances bone neoformation in calvaria bone defects of ovariectomized rats

**DOI:** 10.1590/0103-6440202304980

**Published:** 2023-07-17

**Authors:** Vitória Ricardo, Luiz Gustavo de Sousa, Isabela Hallak Regalo, Dimitrius Leonardo Pitol, Karina Fittipaldi Bombonato-Prado, Simone Cecilio Hallak Regalo, Selma Siessere

**Affiliations:** 1 Department of Basic and Oral Biology, School of Dentistry of Ribeirão Preto, University of São Paulo, Ribeirão Preto, SP, Brazil

**Keywords:** Lycopene, Bone, Ovariectomy, Rats, Antioxidants

## Abstract

Osteoporosis can affect a significant part of the population and fractures are the most common complications associated with this disease, leading to high public health costs. Thus, the prevention of fractures is relevant to individuals with signs and symptoms as well as to the health system. Postmenopausal osteoporosis has been associated with oxidative stress, emphasizing the importance of an efficient defense system to maintain bone health. Lycopene is a carotenoid with antioxidant properties that may stimulate osteoblastogenesis and inhibit osteoclastogenesis. The purpose of this investigation was to analyze the influence of lycopene in the bone neoformation of calvaria defects in ovariectomized rats utilizing the concentration of 45 mg/kg. Wistar Hannover female rats were divided into ovariectomized and sham groups. The ovariectomized animals received 45 mg/kg lycopene (OvxL) or water (Ovx) by daily gavage the day after ovariectomy/sham surgery for 16 weeks. Twelve weeks after ovariectomy, there were performed 5-mm calvaria defects followed by euthanasia after 4 weeks. Samples of bone tissue were collected to perform morphological and morphometrical analysis of the neoformed bone area, and percentage with Software Image J. Morphological evaluation showed mature bone with more osteocytes in the group OVxL when compared to the other groups. The morphometrical analysis demonstrated a significant increase of bone neoformation in the group OvxL (p<0.05). The data obtained suggest that lycopene benefits bone repair in the absence of estrogenic hormones.

## Resumo

A osteoporose afeta grande parte da população e as fraturas são as complicações mais importantes relacionadas a essa doença, gerando altos gastos para o poder público. Dessa forma, a prevenção de fraturas decorrentes da osteoporose torna-se relevante tendo em vista que gera benefícios tanto para o indivíduo acometido pela doença quanto para o sistema de saúde. A osteoporose pós menoupasa tem sido associada ao estresse oxidativo, portanto, um eficiente sistema de defesa antioxidante é primordial para a manutenção da saúde óssea. O licopeno é um carotenoide antioxidante que aparentemente estimula a osteoblastogênese e inibe a osteoclastogênese. O objetivo deste estudo foi analisar a influência do licopeno na neoformação óssea em defeitos de calvária em ratas ovariectomizadas utilizando a concentração de 45 mg/kg. Foram utilizados 15 ratas Wistar Hannover pesando aproximadamente 200g, sendo que 10 animais foram submetidos à ovariectomia bilateral e 5 (Grupo Sham) foram submetidos à simulação da cirurgia de ovariectomia bilateral. Os animais ovariectomizados foram divididos aleatoriamente em 2 grupos: Ovariectomizado (Ovx) e Ovariectomizado Licopeno (OvxL) que receberam água e licopeno respectivamente, por sonda gástrica, diariamente. As administrações iniciaram-se no dia seguinte à cirurgia de ovariectomia e/ou da exposição dos ovários e foram mantidas por 120 dias, data de realização da eutanásia. O grupo *Sham* recebeu água diariamente. Noventa dias após a ovariectomia bilateral foram confecionados defeitos ósseos nas calvárias de todos os animais e após trinta dias as ratas foram eutanasiadas. As amostras de tecido ósseo foram coletadas e foi realizado o processamento para a obtenção das lâminas histológicas. Foram realizadas as análises morfológicas e morfométrica, onde foi estimada a área (mm^2^) e porcentagem (%) relativa de osso neoformado utilizando o Software Image J. A avaliação morfológica evidenciou a ação benéfica do licopeno pois os animais que receberam esse antioxidante apresentaram um tecido ósseo mais maduro, com maior presença de osteócitos quando comparados aos demais grupos. Por meio das análises morfométricas verificou-se maior neoformação óssea para os animais que receberam o licopeno (p<0,05). Diante dos resultados obtidos, concluiu-se que o licopeno na concentração de 45 mg/Kg teve efeito benéfico no processo de reparação, promovendo significante formação óssea frente à ausência de hormônios estrogênicos.

## Introduction

Osteoporosis is characterized by a bone mass decrease, leading to fragility and bone fracture. Its prevalence remains in post-menopause women, although it can occur in the worldwide population with physical, psychological, and financial consequences ^1^. The annual average cost of osteoporotic fracture treatment in Canada, Europe, and the United States is in the range of 5.000 to 6.500 billion dollars, without considering morbidity and loss of productivity. Thus, osteoporosis prevention could reduce costs to the health system ^2^.

Investigations suggest that post-menopause osteoporosis and oxidative stress are closely associated. The imbalance between reactive oxygen species (ROS) and the antioxidant system generates an oxidative stress that might contribute to functional and structural remodeling that favors its occurrence ^3^. Hence, an effective immune system is essential to bone health maintenance, neutralizing oxidants and contributing to activating osteoblast differentiation, as well as the process of mineralization and the reduction of osteoclast activity ^4^.

Lycopene is a carotenoid that contributes to the red color in fruits and vegetables with antioxidant properties. Studies suggest that lycopene may reduce oxidative stress levels as well as the NTx bone resorption marker ^5^. Besides, lycopene promotes an anabolic state in bone metabolism, stimulating osteoblastogenesis and inhibiting osteoclastogenesis. Therefore, it can cooperate for healthy bone tissue, delaying osteolysis ^6^.

Former investigations performed in ovariectomized rats showed positive effects in femoral epiphysis remodeling with low concentrations of lycopene, i.e., 10 mg/kg ^7^. Besides, other authors observed that this carotenoid works in a dose-dependent manner, with higher concentrations (45 mg/kg) being more effective for bone neoformation in long bones than lower ones (15 and 30 mg/kg)^8^.

Osteoporosis can also affect the jaws, which emphasizes the relevance to investigate the effects of lycopene in defects created in bones with distinct embryologic sites from long bones and similar to the jaws ^9^.

Thus, the purpose of this investigation was to evaluate the influence of lycopene in bone neoformation of defects created in the calvaria of ovariectomized rats utilizing the concentration of 45 mg/kg, with the hypothesis that lycopene may enhance bone repair in rats submitted to an experimental model of osteoporosis.

## Material and Methods

Fifteen Wistar Hannover female rats weighing 200 g were utilized after approval from the Ethical Committee for the utilization of animals from FORP/USP (protocol number 2018.1.588.58.9), maintained three in each box, in the bioterium with controlled room temperature, with food and water ‘ad libitum’.

The animals were submitted to two surgical procedures in sequence: bilateral ovariectomy and calvaria bone defects. Both were performed with intramuscular anesthesia of mixed xylazine hydrochloride 2% (10mg/kg, Rompum® - Bayer, Brazil) and ketamine 10% (75mg/kg, Dopalen®, Brazil) after weighing, trichotomy, and assepsy. After surgical procedures, the animals were sutured with silk thread 4.0 (Ethicon Johnson, Brazil) and received a unique dose of intramuscular penicillin G-benzathine antibiotic (Fort Dodge Animal Health®, Brazil) and antiinflammatory Banamine® 0.2mL/100g (Schering-Plough, Brazil).

Bilateral ovariectomy (n=10) was performed by ovary excision and the sham group (n=5) had their ovaries exposed and repositioned in the abdominal cavity. The confirmation of ovariectomy effects was performed by diestrus cycle maintenance and macroscopic exam of atrophic uterine corns.

The ovariectomized (Ovx) rats were randomly divided into Ovx and Ovx+lycopene (OvxL) groups. Lycopene 10% (Galena, Brazil) was diluted in water in the concentration of 45mg/kg ^8^ and daily administered by gavage, beginning the day after ovariectomy/sham procedure for 120 days until euthanasia, which was performed with previous anesthesia (intramuscular anesthesia of mixed xylazine hydrochloride 2% and ketamine 10%) followed by decapitation. Water replaced lycopene in the Ovx and sham groups.

Calvaria bone defects were performed in the left parietal bone with 1mm depth and 5mm diameter with a trephine bur (Neodent, Brazil) at a speed of 3000 rpm and constant irrigation with saline 0.9% ^10^. After 30 days, the rats were anesthetized and bone samples were collected, containing the defect site with a safe margin. The confirmation of ovariectomy was performed through the exam of uterine corns, which were anemic and thin in ovariectomized rats.

For histologic processing, the samples were immersed in formaldehyde 4% for 24 hours, followed by decalcification with EDTA + TRIS 0.5 M changed every 2 days. After 30 days of demineralization, the acid was neutralized with sodium sulfate 5% for 24 hours. The samples were dehydrated in a crescent alcohol series, diaphanized in xylol, and embedded in paraffin. There were performed sample sections with 6-μm thickness stained with Masson trichrome for morphological evaluation. The qualitative analysis was performed with a light microscope (Leica DM 4000B) equipped with a digital camera (DFC310FX, Leica, Germany) to evaluate neoformed bone in the area of calvaria defect, as well as to differentiate preexistent bone in all experimental groups.

Morphometrical analysis was performed utilizing the software Image J to calculate the area (mm^2^) and percentage (%) of neoformed bone utilizing the differential point counting method ^11^, with 40 images per group totalizing 120 evaluates images. Quantitative data presented a normal distribution and were submitted to ANOVA statistical test and Tukey`s test for comparison among groups (p<0.05), utilizing the software GraphPad Prism.

## Results

### Qualitative analysis

**Figure 1:**
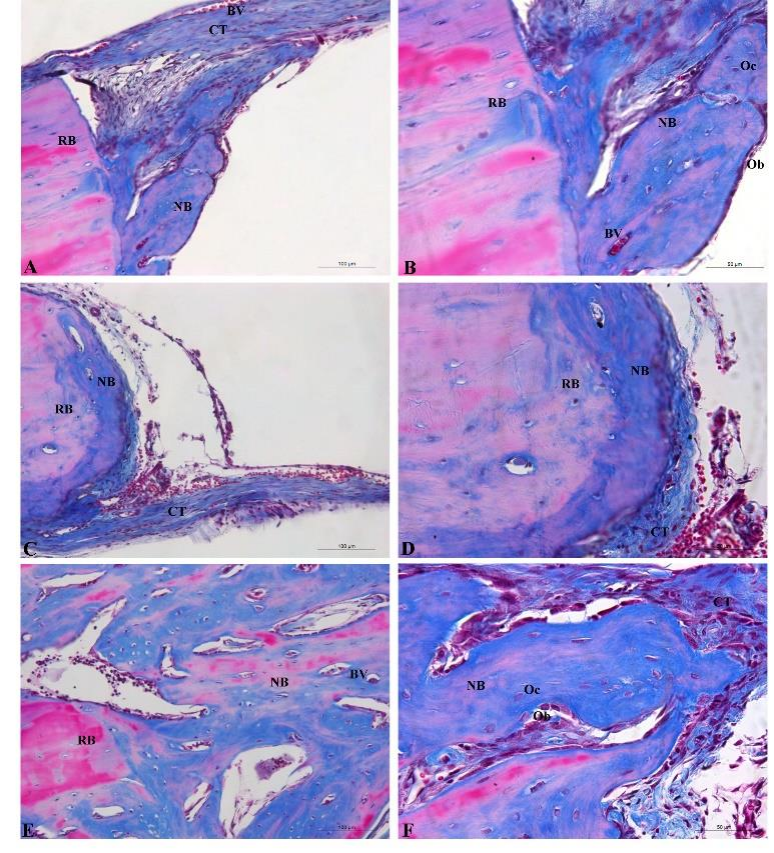
Histological image of calvaria bone defect of the groups Sham (A and B), Ovx (C and D), and OvxL (E and F). RB: remaining bone; NB: neoformed bone; CT: connective tissue; VS: blood vessel; Oc: osteocyte; Ob: osteoblast. Masson’s trichrome staining, scale bar =100 µm.

Figure 1 shows that sham, Ovx, and the experimental group OvxL presented bone neoformation in the borders of the calvaria defect. In the central region of the defect, the connective tissue was thicker in the group OvxL and very thin in the group Ovx, when compared to the control.

There were no inflammatory cells or neoformed bone in the central region of the defect in none of the studied groups. The quantity of neoformed bone in the periphery of the defect was greater in extension in the sham group when compared to the Ovx group. The adjacent connective tissue presented blood vessels, a great number of collagen fibers as well as a great number of fibroblasts. The neoformed bone also presented osteocytes and osteoblasts circumjacent to the matrix.

The group Ovx showed a thin layer of immature neoformed bone with few osteocytes and a thin subjacent connective tissue. The group OvxL was the one with greater bone neoformation, with morphological characteristics similar to the control group with a great number of osteocytes and active osteoblasts in the periphery of neoformed bone. Adjacent to the neoformed bone it was observed an organized connective tissue and characteristics of future differentiation in the new bone matrix.

### Weight gain

**Figure 2:**
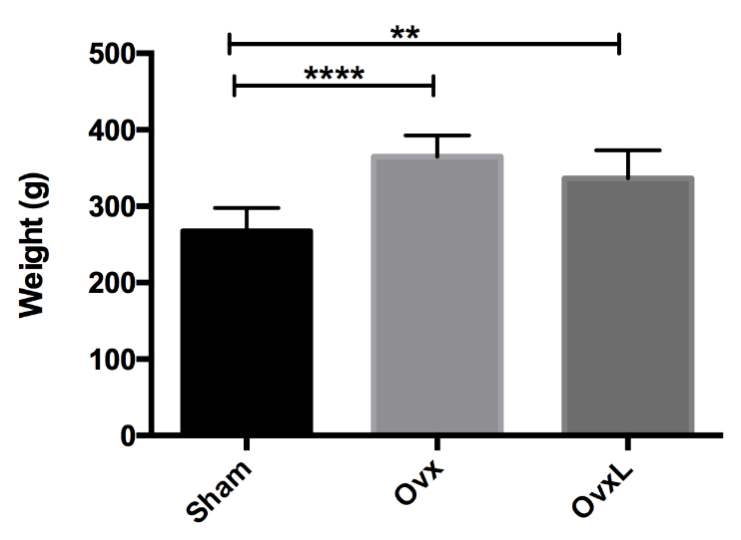
Body weight of animals from Sham, ovariectomized (Ovx), and ovariectomized + lycopene (OvxL) groups.

A post hoc sample size calculation was performed utilizing the values obtained for the neoformed bone area in the groups OvxL (13.52 ± 3.38) and Ovx (5.62 ± 2.48) with an error of 0.05 and a power test of 98.8%, showing that the number of animals was adequate. The Ovx group presented the greater weight gain whereas the sham group presented the lesser weight. The average weight for Sham, Ovx, and OvxL were respectively 267.1±30.5; 364.8±28.0, and 336.6±36.45. Statistical differences were observed between groups Sham and Ovx (p<0.0001) and groups Sham and OvxL (p=0.0036). There was no statistical difference between the groups Ovx and OvxL (p=0.3234) (Figure 2).

### Morphometric analysis of neoformed bone

**Figure 3:**
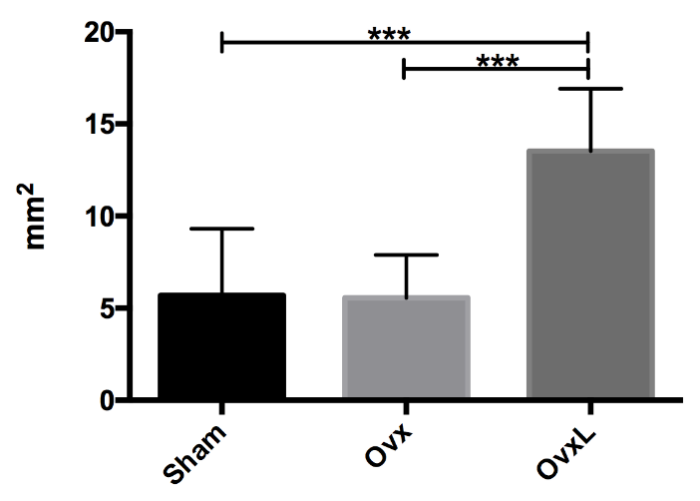
Mean values of neoformed bone area (mm^2^) in Sham, ovariectomized (Ovx), and ovariectomized + lycopene (OvxL) groups.

The evaluation of neoformed bone area (mm^2^) showed that samples from the OvxL group presented the greater bone neoformation whereas the samples from the Ovx group had the lesser formation (Figure 3). The average values for Sham, Ovx, and OvxL groups were respectively 5.69±3.61; 5.62±2.48, and 13.52±3.38. There were differences between Sham and OvxL (p=0.0011) and between Ovx and OvxL (p=0.0010) and no statistical differences between Sham and Ovx (p=0.9989) groups.

**Figure 4:**
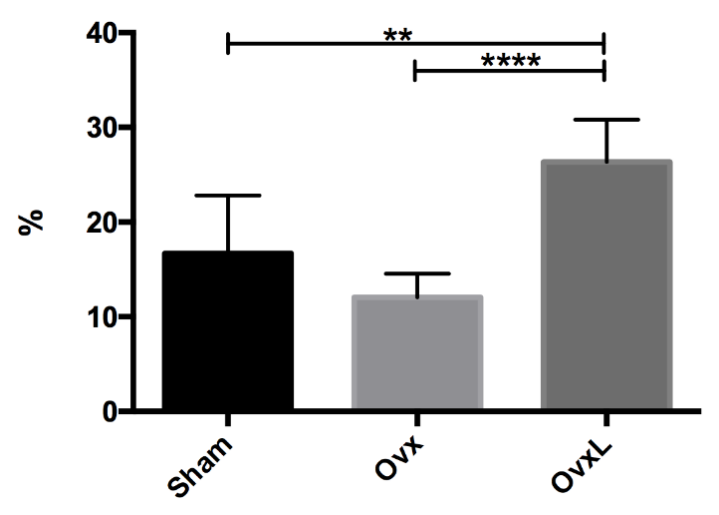
Mean values of neoformed bone percentage in Sham, ovariectomized (Ovx), and ovariectomized + lycopene (OvxL) groups.

The relative percentage of neoformed bone by point counting method (Figure 4), presented the values 16.69±6.12; 12.06±2.49, and 26.36±4.44 for Sham, Ovx, and OvxL respectively. Data presented differences between groups Sham and OvxL (p=0.0038) as well as with groups Ovx and OvxL (p<0.0001). On the other hand, no statistical differences between Sham and Ovx (p=0.1158) were observed.

## Discussion

The present investigation evaluated the effect of lycopene ingestion in the process of bone repair in defects created in the calvaria of ovariectomized rats. The deficiency of estrogenic hormones is associated with bone mass loss, leading to the development of osteoporosis ^12^, simulated with ovariectomy in the present investigation. The reduced estrogenic hormone levels increase the size of osteocyte canaliculi because of nanostructural differences in mineral and matrix levels such as weak collagen fibers, increasing the permeability to small molecules that alter interstitial fluid flow around the osteocytes during mechanical load ^13^.

The ovariectomy model utilized in this investigation follows the protocol of Kalu ^14^, and it has been used to simulate the effects of osteoporosis in women in the post-menopause period. The model of bilateral ovariectomy helps to evaluate bone repair and metabolism with estrogenic hormone deficiency. Nevertheless, the majority of reports utilize long bones such as the tibia and femur ^7^ ^,^ ^15^, with distinct embryologic sites from the jaws ^9^. Hence, the creation of bone defects in the calvaria is relevant for sharing the same embryologic origin of the jaws, and the obtained results might be applied in studies of bone neoformation in this site. Chen et al. ^16^ performed a molecular, cellular, and histological analysis in rat alveolar bone after ovariectomy, observing that its effect is more subtle when compared to long bones. Nevertheless, it is sufficient to delay bone repair in alveolar cavities post-extraction or after osteotomies in extraction sites already repaired. Besides, the same authors indicate that sites under osteotomy presented nonviable osteocytes in ovariectomized groups, associated with more extensive bone remodeling and a delay in osteoblast differentiation. Consequently, the ovariectomy procedure promotes an osteoporotic phenotype that delays the formation of alveolar bone.

Investigations have demonstrated the benefits of lycopene against cancer ^17^ and as an adjuvant in periodontal therapy ^18^. There were no observed positive effects of lycopene associated with weight in the Ovx rats in the present investigation. The literature demonstrates that the deficiency of ovarian hormones induced by bilateral ovariectomy significantly increases body weight ^19^. During the experiments, it was possible to observe that Ovx animals with lycopene intake gained less weight than Ovx animals that received water, but the difference was not significant, in agreement with the reports of Iimura et al. ^20^ and Ardawi et al. ^8^. Despite that, they observed a significant adipose tissue decrease in the animals that received lycopene after evaluation through Dual-energy X-ray absorptiometry (DEXA). Wang et al. ^21^ verified that lycopene supplementation decreased body weight in rats with a diet rich in lipids and fructose, suggesting that lycopene avoids lipidic accumulation through the downregulation of genes associated with lipogenesis and upregulation of genes associated with lipolysis, including functional thermogenic and mitochondrial genes. On the other hand, the animals utilized by Wang et al. ^21^ were males without hormonal disturbances.

The absence of total repair in the defects of the sham group (without ovariectomy and receiving water) in the evaluated period led us to conclude that 5mm-diameter defects are of critical size since the definition of critical size defect depends on the association of the time and repair tissue analysis. Besides, the literature shows that the diameter of 5 mm is suitable to be called a critical size defect, as reported by Hudieb et al. ^22^. The morphological evaluation performed in the present investigation showed a positive effect of lycopene in the bone repair of Ovx rats, promoting a new bone with an abundance of osteocytes compared to the other groups. Our results demonstrate that de novo bone formation was similar in control and ovariectomized groups, in agreement with former reports ^23^. Yu et al. ^24^ suggest that this capacity of bone neoformation in the presence of osteoporosis similar to a healthy condition might be a consequence of great metabolic energy from an increase of ATP generation immediately after ovariectomy.

The ovariectomized group that received lycopene presented a greater area of neoformed bone, confirming our hypothesis. These results are in agreement with Li et al. ^15^, which also verified an increase in bone neoformation around implants in the femur of osteopenic female rats that received lycopene. These same authors observed that lycopene enhances osseointegration and fixation of the implants, suggesting that this antioxidant might be a promising therapeutic agent to avoid bone loss and delayed osseointegration in osteopenic conditions.

The increased bone formation promoted by lycopene intake might be explained by its cellular and molecular effects on osteoblast and osteoclast differentiation. Lycopene decreases osteoclastogenesis and increases osteoblastogenesis by apoptosis inhibition, promoting significant changes in the MEK signalization pathway. Besides, it is suggested that lycopene might affect the kinase C protein pathway in osteoclasts and NFkB signalization in osteoblasts ^6^. Russo et al. ^25^ also observed that lycopene might enhance osteoblast metabolism and influence its differentiation and synthesis of collagen. Besides, lycopene inhibits RANKL expression, indicating a role in the suppression of bone resorption. The increased bone neoformation might also be explained by its capacity to upregulate the expression of Sp7, Runx2, Bsp, and Bglap genes, as observed by Oliveira et al. ^7^, in osteoblasts of ovariectomized rats. The same investigation demonstrated that daily ingestion of 10 mg/kg lycopene for 60 days diminished bone loss in the femoral epiphysis of ovariectomized rats, with values similar to the control.

The results obtained in the present investigation suggest that lycopene in the concentration of 45 mg/Kg enhanced the process of bone repair, promoting significant bone formation in the absence of estrogenic hormones. More studies are needed to verify this concentration at cellular and molecular levels.
